# The interface between rare plant management and genetics: conserving Pitcher’s thistle (*Cirsium pitcheri*) in the Great Lakes region, USA

**DOI:** 10.1093/aob/mcaf207

**Published:** 2025-10-22

**Authors:** Noel B Pavlovic, Jeremie B Fant, A Kathryn McEachern

**Affiliations:** U.S. Geological Survey, Great Lakes Science Center, Lake Michigan Ecological Research Station, Chesterton, IN 46304, USA; Negaunee Institute for Plant Conservation Science and Action, Chicago Botanic Garden, Glencoe, IL 60022, USA; U.S. Geological Survey, Western Ecological Research Center, Channel Islands Field Station, Ventura, CA 93001, USA

**Keywords:** Reintroduction, founder population, *Cirsium pitcheri*, Pitcher’s thistle, Great Lakes, endemic, sand dunes, seed, genetics, conservation, monocarpic perennial

## Abstract

**Background and Aims:**

Tools showing best conservation practices are becoming critical for rare plant conservation as populations become isolated through habitat fragmentation and changing ecosystem processes. We illustrate the importance of reintroduction and assisted gene flow, using reintroduction of the USA federally threatened Pitcher’s thistle (*Cirsium pitcheri*), a monocarpic perennial endemic to the western Great Lakes sand dunes. We evaluate the success of experimental reintroductions of this species into its dynamic coastal environment and address fundamental issues for rare plant reintroduction, including questions of maintenance of genetic diversity, founder size, introduction methods and assisted gene flow effectiveness.

**Methods:**

In 1994 we initiated experimental reintroductions at three new locations along a habitat successional gradient using 4200 seeds collected from 54 maternal lines. At each site, seed sources were distributed among replicate blocks that were split by sowing method (sowing vs broadcasting) to examine establishment success. We monitored population demography for 30 years, assessing genetic variation in 2009 and regionally from 1997 to 2014.

**Key Results:**

Two of three populations persisted for 30 years from a single-seeding founder event. Reintroduction populations had greater expected heterozygosity than regional native populations. Despite persistence, moderate inbreeding coefficients showed that reintroduction populations have not achieved an effective size to reduce the likelihood of inbreeding depression. However, the mid-successional reintroduction had the lowest kinship of all populations sampled, indicating a healthy restoration. Seed sowing produced three times as many seedlings as broadcasting, but seed source did not affect germination success. Persistence has been facilitated by local migration to suitable habitat patches, an important metapopulation process.

**Conclusions:**

Seeds can be an effective method for reintroducing monocarpic plants in high-quality habitats. However, low numbers of reproductive adults and moderate inbreeding illustrate the need for repeated additions of plants or seed to reduce inbreeding to improve rare plant genetic evolutionary potential.

## INTRODUCTION

As human impacts on ecosystems increase (Moreira *et al.*, 2023) and habitats shift due to changing climate, development of rare plant conservation strategies is becoming ever more critical in stemming the tide of species losses (Fenster *et al.*, 2018; Ralls *et al.*, 2018; Frankham *et al.*, 2019; Kress and Krupnick, 2022), especially in unique habitats with endemic species (Tietje *et al.*, 2023). Plant reintroduction, a central strategy for plant conservation, has flourished as indicated by national and multinational databases for tracking rare plant reintroduction efforts (Fenu *et al.,* 2023; Bellis *et al.*, 2024; Godefroid *et al.*, 2025) and elucidating pitfalls and successes in rare plant reintroduction (Bellis *et al.*, 2023, 2025). Critical to these conservation efforts are identification of high-quality habitats that can maintain positive population growth (Knight, 2012) and introduction of high genetic variability for sustained evolutionary potential (Frankham *et al.*, 2017). Translocations or reintroductions of rare plant populations can be important tools for improving species long-term persistence by either creating larger or multiple populations or increasing inter-population connections (Bellis *et al.*, 2023). In the USA, this has become an important component of endangered species recovery plans, which often call for establishment of self-sustaining populations in new sites or in sites where populations have been extirpated, but they rarely address genetic conservation topics (U.S. Fish and Wildlife Service, 2002; Pierson *et al.*, 2016). Given that the need for reintroduction activities is increasing at a pace faster than the science needed to plan these activities, we should frame restorations in an experimental context (Suding, 2011; Whitehead *et al.*, 2023). This approach can inform our understanding of the species’ ecological and genetic factors affecting recovery prospects while actively helping the recovery of the species *in situ* (Maschinski *et al.*, 2023).

Many factors determine an out-planting strategy and aid in understanding outcomes. Factors extrinsic to the species include habitat quality and extent, prospects for amelioration of stressors and threats, management activities and the commitment to long-term protection (Maschinski and Albrecht, 2022). However, there are also many intrinsic factors that inform translocation success. Many of these require making important genetic decisions, including selection of source and numbers of founding individuals for a reintroduction. This also requires identifying those source populations that produce sufficient seeds, are genetically diverse, adapted to local conditions and have limited evidence of inbreeding depression, which is a finite/fixed resource in the conservation effort. Aside from the genetic makeup of the individuals, other factors affecting success including life-history, breeding system, niche requirements (Duminil *et al.*, 2007; Angeloni *et al.*, 2011) and plant stage and method of reintroduction (sowing, broadcasting, transplanting) (Bellis *et al.*, 2023).

To further the development of rare endemic plant conservation, we present a retrospective analysis of a 30-year (about six generations) reintroduction experiment of a Great Lakes sand dune endemic monocarpic perennial, Pitcher’s thistle (*Cirsium pitcheri*). This thistle is found across a plant community successional gradient in sparsely vegetated dune habitats along the sandy shores of Lakes Michigan, Superior and Huron (Loveless, 1984; Nantel *et al.*, 2018; Morris *et al.*, 2023). It is a colonizer of newly exposed early-successional dunes, gradually declining locally as vegetation density increases and progresses from mid- to late-successional stages (McEachern, 1992). Consequently, this thistle persists as metapopulations with many small ephemeral subpopulations within larger dune landscapes, constantly colonizing new sites as they open through natural dynamics related to shifts in lake levels driven by continental-scale climate and wind patterns (McEachern *et al.*, 1994).

Given declining numbers of plants and subpopulations within the southern parts of the range, efforts to reintroduce and expand numbers of Pitcher’s thistle populations were initiated in the 1990s at Illinois Beach State Park (SP) in Illinois (Bowles *et al.*, 1993; Bell *et al.*, 2002) and in this project in Indiana, USA. At project inception in 1994, we knew that the species had low genetic diversity, based on allozyme analyses by Loveless and Hamrick (1988), and that plants could self-pollinate but outcrossing increased seed set by ∼16-fold, from 4.6 to 74.8 % in *ex situ* experiments (Loveless, 1984). Therefore, we reasoned that using seeds from several native populations collected from a range of successional habitats would increase genetic diversity in out-planted sites, ultimately resulting in more resilient populations (Soule, 1986 ; Pavlik and Barbour, 1988; Maunder, 1992). We established a programme of long-term demography studies (McEachern *et al.*, 1989) and periodic genetic monitoring (Fant *et al.*, 2013, 2014) to evaluate the progress of the restorations *in situ*, and modelled metapopulation dynamics (Halsey *et al.*, 2015, 2016) and range-wide genetic structure (Fant *et al.*, 2014) to infer contributions of the local introductions to regional and continental species persistence and diversity. We also hypothesized that Pitcher’s thistle is a species that exhibits metapopulation dynamics and that populations would move around in the landscape as suitable habitat changed with changing dune processes (McEachern *et al.*, 1994).

Our goals in this paper are to address the following questions: (1) Did we successfully develop a viable self-sustaining population with a one-time founder event? (2) Does broadcasting seeds produce more seedlings than sown seeds? (3) Did different seed sources establish differentially at common sites and do they have implications for restoration demography? (4) Did restorations expand spatially in the successionally dynamic landscape? (5) Does the introduction of new populations increase metapopulation viability? (6) Can we stave off the effects of inbreeding depression by establishing a genetically diverse restoration?

## MATERIALS AND METHODS

### Study species

Pitcher’s thistle (*Cirsium pitcheri*) inhabits a latitudinal range spanning nearly 760 km from the south shore of Lake Michigan to the south shore of Lake Huron and southern and eastern shores of Lake Superior (Nantel *et al.*, 2018; U.S. Fish and Wildlife Service, 2002). It was listed as federally threatened in 1988 (Harrison, 1988), because shoreline use was fragmenting and damaging habitats, and populations were shrinking and disappearing at a rapid rate (listed as vulnerable, G3, by NatureServe, 2024). At the southern extent of its range along the south shore of Lake Michigan in Indiana, populations were protected in the Indiana Dunes SP when it was created in 1926 (Cottman, 1930) and at Indiana Dunes National Park (INDU) in 1967 (Cockrell, 1988).

**Fig. 1.**
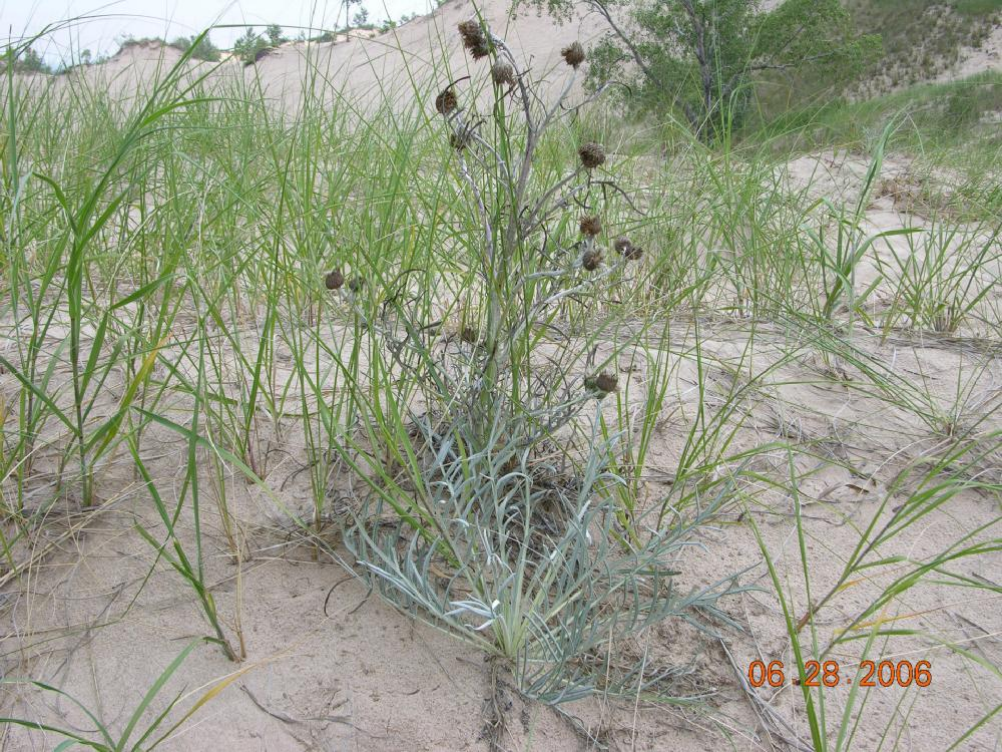
Pitcher’s thistle (*Cirsium pitcheri*) flowering adult and juvenile plants, Indiana Dunes National Park, Indiana, USA. Photo credit: A. K. McEachern.

Pitcher’s thistle produces cream to light pink-coloured flowers on a blue–grey statuesque stalk at ∼3–8 years of age; after flowering the plant dies (Fig. 1) (Loveless, 1984; McEachern 1992; Priemer and Girdler, 2024). Thus, plants must survive for 3–8 years, the time it takes to accumulate resources to reach a root crown diameter greater than ∼12 mm (McEachern, 1992), before reproduction and potential for population increase. Pitcher’s thistle is self-compatible and has a mixed-mating breeding system, as Loveless (1984) found, with outcrossing rates of 35–88 %, thus demonstrating that intra- or inter-head geitonogamy is possible (several heads on a plant are in bloom at the same time). Flower heads have tubular bisexual flowers that mature centripetally; thus, the head is male on the first day of blooming and wholly female by the fourth day of blooming, resulting in intra-head herkogamy and partial dichogamy (Loveless, 1984). Since pollen dispersal requires insect visitation, this flowering sequence increases the probability of cross-pollination by generalist bees and flies by pollen deposition from other flowers on the stigmas (Loveless, 1984). Pitcher’s thistle does not spread by vegetative growth; instead, it depends solely on seed production for establishment of new plants. The seeds, the largest among thistles in eastern North America, germinate readily (McEachern, 1992; Maun, 1996) and their germination success is dependent on genetic source, seed storage environment, seed size and depth of seed burial (Hamze and Jolls, 2000) and absence of litter cover (Jolls *et al.*, 2015). Research shows that seed production is reduced by several native insects (*Platyptilia carduidactyla* and *Baris subsimilis*) and greatly reduced by non-native seed-feeding weevils (*Larinus carlinae* and *Rhinocyllus conicus*) and one root-boring weevil (*Cleonus pigra*), with the potential to drive local populations to extinction (Bevill *et al.*, 1999; Havens *et al.*, 2012). This pattern varies across the thistle’s latitudinal range, with the greatest effects in late-successional habitats where litter is abundant (Havens *et al.*, 2012; Jolls *et al.*, 2015; Hakes and Meunier, 2018).

### Study location

The inland freshwater coastal dunes of the US and Canadian Great Lakes are a site of regional plant endemism (Guire and Voss, 1963; Pierson, 2010). The southern dunes of Lake Michigan occur as long linear beach-level early-successional foredunes extending 24 km (15 miles) along the coastline, punctuated by enormous dune blowouts that form during lake-level high stands when north-west winds and waves breach the foredunes and push open sand inland up to 0.5 km (0.3 mile) through forest into parabolic dunes reaching as high as 58 m (92 feet) above lake level (Cowles, 1899; Tansley, 1913; Olson 1958*b*; Thompson, 1987, 1994; Chrzastowski *et al.*, 1994; Loope and Arbogast, 2000). Lake Michigan water levels fluctuate from high to low in climate-driven cycles, historically occurring in periods varying from 35 to 150 years (Baedke and Thompson, 2000; Wilcox *et al.*, 2007; Fisher *et al.*, 2021). As lake levels recede, beach sand is exposed, wind moves sand inland from the widened beach, rebuilding the foredunes. Earlier, Cowles (1899) and Olson (1958*a*) noted that such periodic rises in lake level eliminated foredunes, requiring vegetation recolonization from the interior blowouts as lake levels receded and foredunes rebuilt. Most recently, Lake Michigan water levels reached high stands in 1986–87 (Sellinger *et al.*, 2008) and 2019–20 (Theuerkauf and Braun, 2021), virtually eliminating the foredunes and leaving the remaining natural blowouts as the sole habitat for Pitcher’s thistle at the southern extent of its range (McEachern *et al.*, 1989; Vitt *et al.*, 2024).

### Origins and genetic structure

Pitcher’s thistle is believed to have descended from *Cirsium canescens* (Platt thistle), a Nebraska sandhills endemic species (Loveless and Hamrick, 1988), during the late Pleistocene because of isolation in the Great Lakes area as glaciers expanded and receded, creating sandy shoreline habitats. Range-wide genetic study (Fant *et al.*, 2014), suggests that Pitcher’s thistle migrated back into its current range from the north-east where genetic diversity is highest, consistent with this area being on the trailing edge of a species distribution (Petit *et al.*, 2003; Hampe and Petit, 2005). Although genetic diversity is low across all Pitcher’s thistle populations, the southern and western populations have the least diversity, consistent with being on the leading edge of range expansion (Hampe and Petit, 2005), with some evidence that these populations are recent arrivals because of lake-level changes that occurred 7000 years ago (Colman *et al.*, 1994). Indeed, the configuration and dynamics of the northwest Indiana dune system suggest that Pitcher’s thistle has persisted there as a metapopulation inhabiting a series of massive inland dune blowouts connected by intermittently stable linear foredunes running along the beach between blowouts (McEachern *et al.*, 1994). The low diversity and high isolation in southern populations is consistent with repeated bottlenecks from populations establishing from a relatively small number of seeds (Loveless and Hamrick, 1988; Fant *et al.*, 2014), which is supported by studies of reintroductions (Fant *et al.*, 2013) where recruits in sub-populations have infrequent to non-existent immigration of new propagules (Fant *et al.*, 2013). Metapopulation dynamics models constructed in 2016 (Halsey *et al.*, 2016) from our long-term native and introduced population demography data demonstrate relative contributions to metapopulation functioning by both native and restored populations in the Indiana Dunes system.

### Site selection

**Fig. 2.**
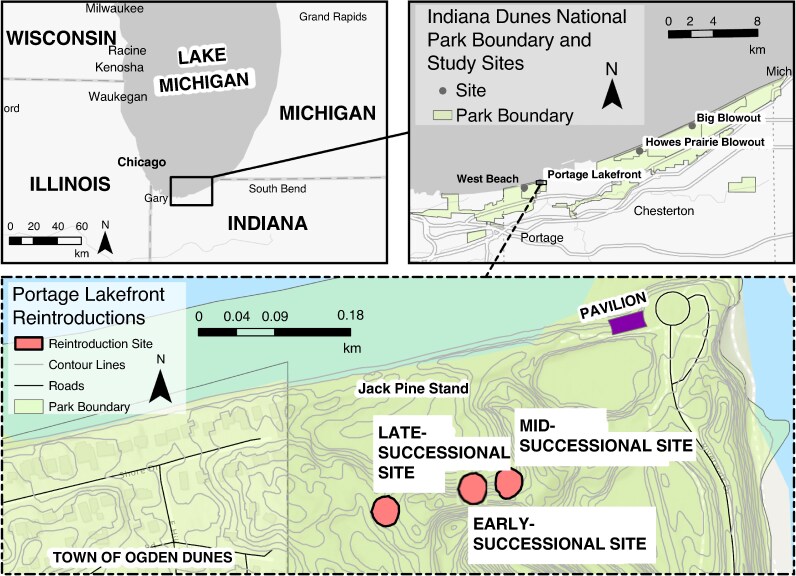
Maps showing the location of the study site in the Great Lakes region (top left), locations of seed collection sites within the Indiana Dunes National Park, IN, USA (top right) and locations of reintroduction sites in the Portage Lakefront blowout (bottom).

**Fig. 3.**
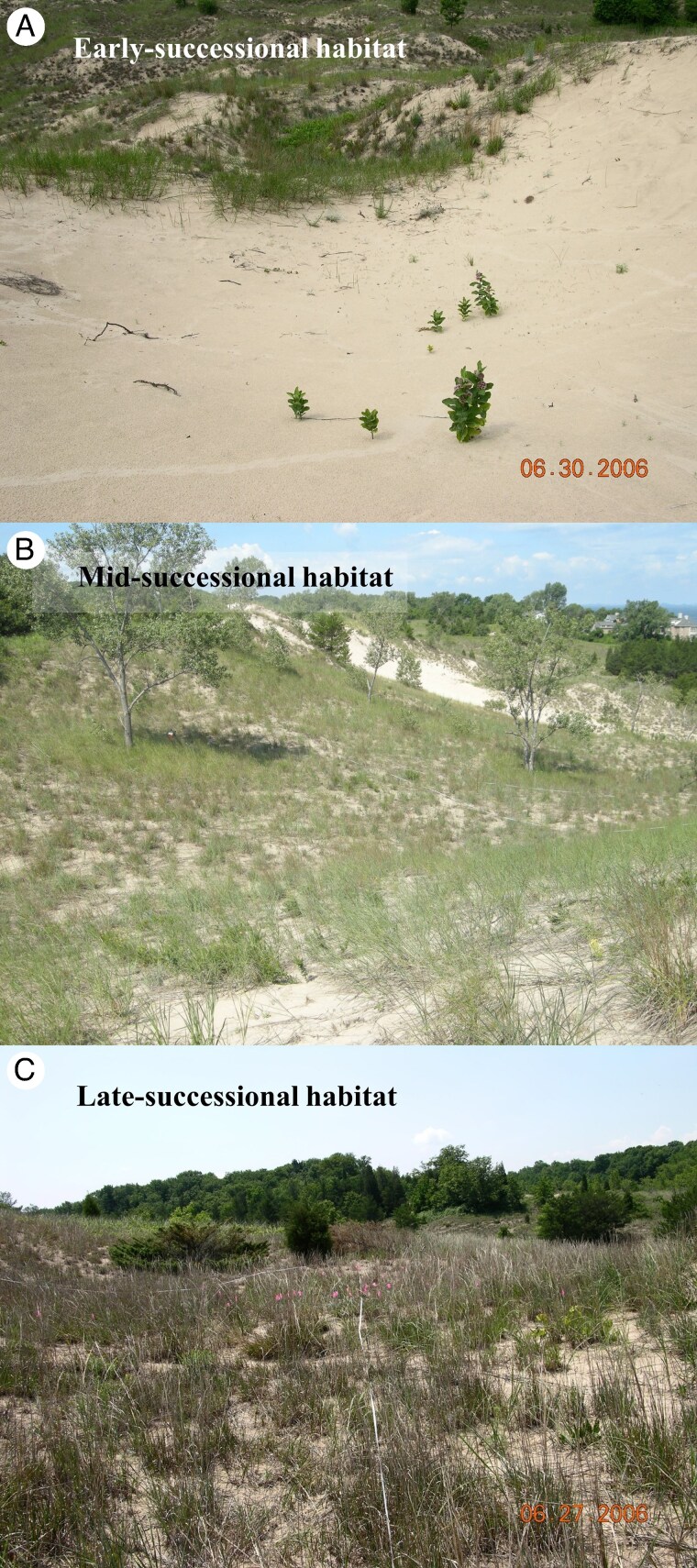
Pitcher’s thistle (*Cirsium pitcheri*) reintroduction sites illustrating the successional habitat gradient: early-successional (bare sand) site (A), mid-successional (marram grass) site (B) and late-successional (little bluestem) site (C); Indiana Dunes National Park, IN, USA. Photo credit: A. K. McEachern.

Our long-term goal was to mimic natural thistle population dynamics by introducing seeds to a blowout in a one-time simulated artificial founder event. We planted in the Portage Lakefront blowout near the town of Ogden Dunes, located near the centre of the Indiana Pitcher’s thistle distribution (Fig. 2). The shoreward mouth of the blowout had a low foredune with sand colonized by the pioneering rhizomatous marram grass (*Ammophila breviligulata*), surrounding a small wetland panne in the early stages of colonization by Jack pine (*Pinus banksiana*). Grass density increased inland to a mid-successional mix of marram grass, sand reed grass (*Calamovilfa longifolia*) and little bluestem (*Schizachyrium scoparium*). Farther inland, this secondary dune was being colonized by common juniper (*Juniperus communis*) and Jack pine (*P. banskiana*), in a pattern of classic dune succession (Cowles, 1899). The three planting locations, across a successional gradient, were an early-successional bare sand blowout (Fig. 3A), a mid-successional blowout valley dominated by marram grass (formerly called Ogden Dunes East (Halsey *et al.*, 2015)) (Fig. 3B) and a late-successional secondary dune crest dominated by little bluestem grass (Ogden Dunes West (Halsey *et al.*, 2015)) (Fig. 3C) (hereafter sites are referred to as early-, mid- and late-successional). Distances among plots were approximately 45 m for early- to mid-, 150 m for mid- to late- and 107 m for early- to late-successional sites.

### Seed sources and plot design

Seeds were collected on 4 July and 11 August 1993 from the three largest Indiana Pitcher’s thistle blowout populations, including West Beach, 2.2 km to the west of the reintroduction site, 14 maternal lines; Howes Prairie, 9.2 km to the east, 20 maternal lines; and Big Blowout, 14.5 km to the east, 20 maternal lines (mean distance = 8.6 ± 2.9 km (s.e. throughout)) (Fig. 2). These sites represent some of the highest diversity and moderate to low inbreeding within the southern region (Fant *et al.*, 2014). These 54 maternal plants inhabited habitat patches representing a mix of successional stages ranging from early- (22%) to mid- (55%) and late-successional (24%) species composition. At West Beach the maternal plants were the most highly dispersed, averaging ∼50 m from a central survey conduit, while at both Howes Prairie and Big Blowout they averaged 35 m from the conduit. Collections were taken from among the terminal one to four heads of each plant, averaging ∼77 seeds per parent. The collections were spread evenly among flowering individuals, taking less than ∼10 % of the seeds from any one individual (Elliott *et al.*, 1999; Maschinski and Albrecht, 2017). We estimated that we collected from ∼25 % of the flowering plants at West Beach, 18 % at Howes Prairie and 29 % at Big Blowout.

Seeds were sorted in the laboratory and counted according to apparent quality: ‘perfect’ seeds were completely filled and uniformly medium tan and shiny; ‘marginal’ seeds were not completely full and they compressed somewhat when pressed gently with a fingernail and were slightly smaller, but still brown and shiny. We obtained a total of 3600 perfect and 600 marginal seeds, totalling 4200. An even number of seeds from each maternal line was selected for each reintroduction site (Table 1). A total of 1200 perfect seeds were planted at the early- and the mid-succession sites, with an even mix of 400 seeds from each of the three source populations. We planted 1800 seeds at the late-successional site, including 400 perfect seeds from each source and 200 marginal seeds from each of the source populations (Table 1).

**Table 1.** Source populations, number of maternal lines and quantity of seed used in early-, mid- and late-successional reintroduction sites at Indiana Dunes National Park, IN, USA in 1994.

Donor site	Maternal lines collected	Early-successional perfect seeds	Mid-successional perfect seeds	Late-successional perfect seeds	Late-successional marginal seeds
West Beach	14	400	400	400	200
Howes Prairie	20	400	400	400	200
Big Blowout	20	400	400	400	200
Total	54	1200	1200	1200	600

**Fig. 4.**
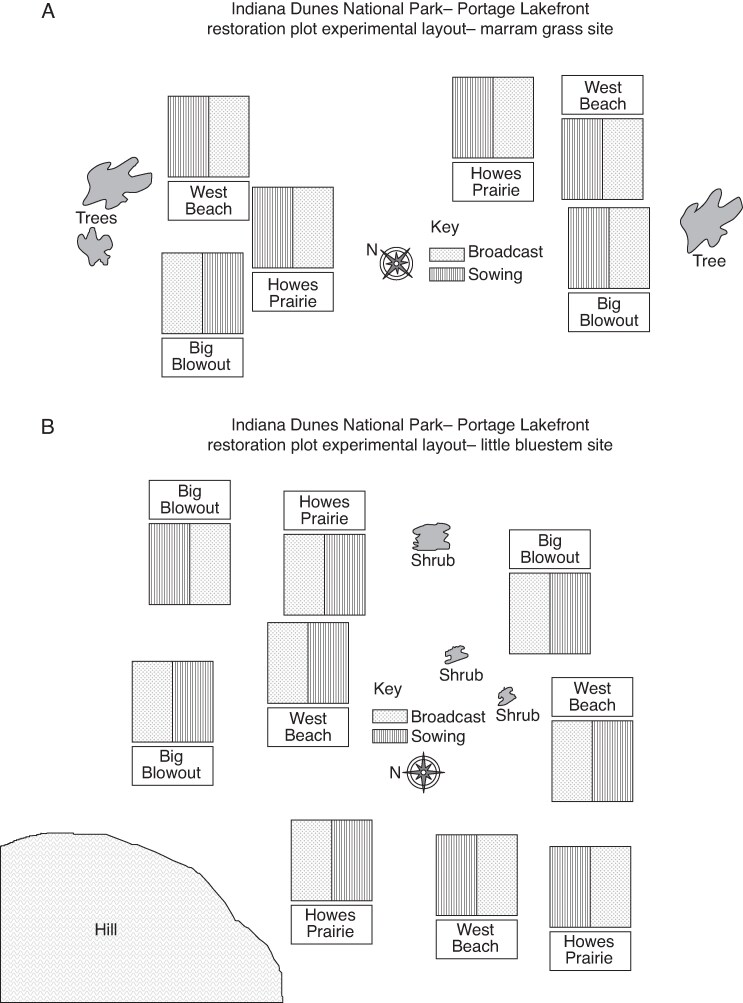
Split-plot planting design at Pitcher’s thistle (*Cirsium pitcheri*) reintroduction sites in the Portage Lakefront blowout at the Indiana Dunes National Park, IN, USA. (A) Mid-successional (marram grass (*Ammophila breviligulata*)) site. (B) Late-successional (little bluestem (*Schizachrium scoparium*)) site. The early-successional site is not shown.

We planted seeds on 14 May 1994 in a split-plot design (Fig. 4) at each site to test the effect of seed source and planting method; each split plot received 100 seeds. We planted six 5 × 5-m blocks in early- and mid-successional habitats each, and nine blocks in the late-successional dune grassland. Each block was assigned a seed source and seed type, and blocks were split randomly into sowing (manually planted at 1 cm sand depth) and broadcast (scattered on the ground surface) treatments. At the late-successional site, the perfect and marginal seeds were sown in separate blocks.

### Sampling design

We revisited each site annually in mid- to late-June to count plants by blocks (1995–2000) and measured individuals (2001 onwards) as seedlings (plants with current year cotyledons), juveniles (non-flowering rosettes) and adults (bolted flowering plants). After 2000, a steel conduit was pounded into the ground in the centre of the planting area, and the distance and azimuth to each plant were measured from that conduit up to 17.84 m (1/10th circular hectare). For each plant, we recorded life stage, numbers of leaves and root crown diameter for juveniles and adults, and when plants flowered we counted numbers of inflorescences (Pavlovic and McEachern, 2025a; https://doi.org/10.5066/P13KOGHK). As Pitcher’s thistle seeds migrated outside the plot, we increased the plot size to capture those new plants. To examine temporal changes in genetic variation of reintroduced and native populations, we used genetic data from three sources: (1) Fant *et al.* (2013), (2) unpublished genetic variation data collected by J.B.F. and Kayri Havens from 1997 and 2003 from the same Fant *et al.* (2013) sites, and (3) 2014 genetic variation from Sefton (2020) collected at two Michigan native populations that were sampled previously by Fant *et al.* (2013). Fant *et al.* (2013) and Sefton (2020) sampled respectively 50 and 20 individuals per site and six and eight microsatellite loci to examine genetic variation among sites. Both genetic diversity (*H*_e_) (Fant *et al.*, 2013; Sefton, 2020) and kinship coefficients (not previously published) (Queller and Goodnight, 1989) within populations were calculated using Genalex 6.5 (Peakall and Smouse, 2006).

### Statistical analysis

Two-way split plot mixed effects ANOVA was used to examine the effects of provenance and sowing method on the germination of Pitcher’s thistle for 1995, 1996 and sum of the two years. We used a three-way split-plot ANOVA on the late-successional germination data to examine effects of provenance, sowing method and seed condition (Box *et al.*, 1978; Devore, 1982). Tallies of seedling, juveniles and adult plants were summed to give totals by stage (census *N* = *N*c) per year for the early-, mid-, and late-successional plots. Results are presented as means plus or minus the standard error.

## RESULTS

### Seed germination

Pitcher’s thistle seedlings did not appear in 1994, but they were present in all three sites in 1995 (Table 2). Overall, 6, 37 and 16 % of the seeds germinated in 1995 respectively at the early-, mid- and late-successional sites. The early-successional plot was buried by ∼1 m of sand that blew in with the 1995–96 winter storms and has remained covered in sand with no Pitcher’s thistle plants thereafter. Seedlings germinated in the mid- and late-successional sites again in 1996, two seedlings were present in the late-successional site in 1997, and one seedling appeared in 1999 (Table 2). At the mid-successional site, seedling numbers were greater in 1995 than in 1996 (448 vs 11, respectively); while seedling numbers were more nearly equal at the late-successional site 1995–96 (287 vs 346, respectively). Numbers of plants declined precipitously after initial flushes of germination in 1995 and 1996, at both the mid- (90 % decline from 1995 to 1996) and late-successional sites (68 % from 1996 to 1997) (Table 2).

**Table 2.** Initial seeds, seedlings, juveniles and reproductive adult plants at three Pitcher’s thistle (*Cirsium pitcheri*) planting sites 1994–2000 in the Portage Lakefront blowout at Indiana Dunes National Park, IN, USA. Sites are early- (dominated by bare sand), mid- (marram grass) and late-successional (little bluestem grass). Seed counts for 1994 are the total seeds sown at each site.

Sites	Stage	1994 seeds	1995	1996	1997	1998	1999	2000
Early-successional	Seedlings	1200	65	0	0	0	0	0
Mid-successional	Seedlings	1200	448	11	0	0	0	0
	Juveniles	−	0	36	40	47	44	25
	Adults	−	0	0	0	0	3	12
Late-successional	Seedlings	1800	287	346	2	0	1	17
	Juveniles	−	0	52	126	114	99	56
	Adults	−	0	0	0	0	5	28

Sowing produced three to five times more seedlings than broadcasting at both sites with combined 1995 and 1996 seedling counts (mid-successional: *F*_1,3_ = 11.96, *P* = 0.014; late-successional: *F*_1,3_ = 45.35, *P* = 0.001: 53 ± 6 seedlings > 17 ± 3 seedlings for 2-year sum). Interestingly, the opposite was true at the early-successional site: about six times fewer seedlings resulted from sowing than from broadcasting there. There was no significant effect of seed source on germination (early-successional: *F*_2,12_ = 3.12, *P* = 0.185; mid-successional: *F*_2,12_ = 1.09, *P* = 0.44; late-successional: *F*_2,12_ = 5.44, *P* = 0.101).

### Population persistence

**Fig. 5.**
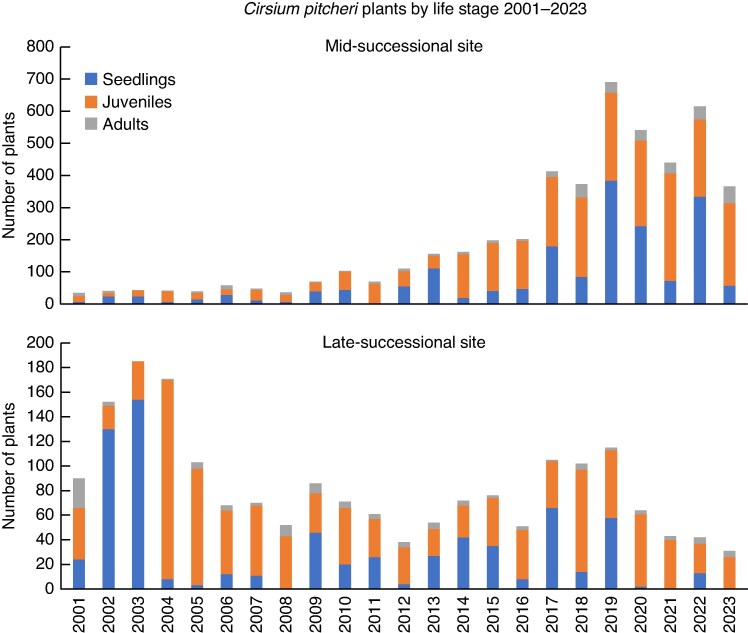
Change in Pitcher’s thistle (*Cirsium pitcheri*) population size by life stage class from 2001 to 2023 at the mid-successional (marram grass (*Ammophila breviligulata*)) and late-successional (little bluestem (*Schizachyrium scoparium*)) reintroduction populations at Indiana Dunes National Park, IN, USA. The top of the stacked life stage bars shows the total population size for that year. Note the *y* axis for the late-successional plot is a quarter of that for the mid-successional graph.

The mid- and late-successional populations existed as collections of juvenile plants until the first flowering event in 1999 (Table 2), hovering at about 50 juveniles at the mid-successional site and around 100 juveniles at the late-successional site. The first cohorts of seedlings occurred in 2001 in the mid-successional site and in 1999 in the late-successional site, respectively 7 and 5 years from the start of the experiment (Fig. 5 and Table 2). Both reintroductions have persisted for the full 30 years (1995–2023) without additional seed augmentation, with highly variable numbers of plants driven by episodic fluctuation in seedling recruitment (Fig. 5). Population sizes averaged 210 ± 207 and 83 ± 41 respectively for the mid- and late-successional sites. Seedlings have ranged from 3 to 71 % of the total population at the mid-successional site and from 0 to 85 % at the late-successional site, while flowering adult plants varied from 2 to 26 and 0 to 27 % at the two sites respectively. The mid-successional population showed an episodic population peak in 2019, spanning 7 years from 2017 to 2023, a phenomenon initiated but not sustained in the late-successional site. In 2009, the year of genetic sampling, the mid- and late-successional populations had respectively 66 plants (38 seedlings, 26 juveniles and 2 adults) and 83 plants (45 seedlings, 30 juveniles and 8 adults). In 2023, the mid- and late-successional sites respectively had population sizes that were 31 and 2 % of the founding number of seeds planted in 1994, and 79 and 5 % of the founding seedlings establishing in 1995–99 (Table 2).

### Genetic makeup

**Fig. 6.**
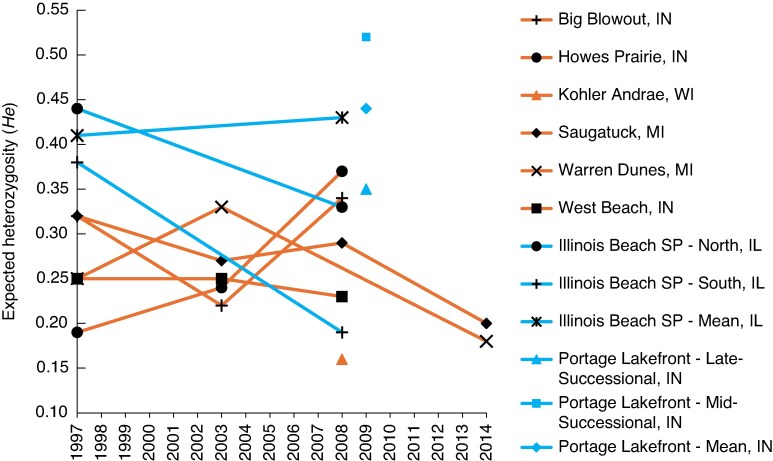
Temporal changes in expected heterozygosity (*H*_e_) among native and introduced populations of Pitcher’s thistle (*Cirsium pitcheri*) in Illinois, southern Indiana and southern Michigan and Wisconsin from 1997 to 2014. Native populations have orange lines or symbols and reintroduction sites have blue lines or symbols.

With the addition of samples from other time periods from Sefton (2020) and J.B.F. (unpublished data from 1997 and 2003) expected heterozygosity (*H*_e_) continued to be greater in individual reintroduction populations compared with native populations, as shown in the mean lines for Illinois Beach SP and mean values for Portage Lakefront populations (Fig. 6 and Table 3). Overall, reintroductions had higher average *H*_e_ than native populations (reintroductions = 0.37 ± 0.05, native = 0.26 ± 0.01; *t*_16.9_ = −11.2, *P* < 0.001), with a few exceptions. These were Illinois Beach SP North and South populations, where *H*_e_ declined below the observed native maximum value of 0.37 (at Howes Prairie, IN) by 2008 and in 2009 at the late-successional population at Portage Lakefront (Table 3, Fig. 6). The native populations show considerable variation in time and among sites (Fig. 6). Sefton’s (2020) resampling of native populations at Warren Dunes SP and Saugatuck Dunes SP 6 years later in 2014 show declining genetic diversity at Warren Dunes SP and Saugatuck Dunes SP (*H*_e_ decreased from 0.33 to 0.18 and 0.32 to 0.20 respectively), along with increasing inbreeding coefficients at both sites (0.16–0.54 at Warren Dunes SP and 0.19–0.23 at Saugatuck Dunes SP) (Table 3 and Fig. 6).

**Table 3.** Population and genetic data for native and reintroduced pitcher’s thistle (*Cirsium pitcheri*) populations in southern lake Michigan, based on respectively six (Fant *et al*., 2013) and eight (Sefton, 2020) polymorphic microsatellite loci. Status indicates whether the population is native or reintroduced. Population sizes are the estimated size of the total number of plants in the population at the time of sampling (Fant *et al.*, 2014): 5 = <100, 4 = 100–500 and 3 = 500. Average sampling effort is the number of plants sampled. *H*_e_ is genetic diversity, *F*_IS_ is Wright’s coefficient of inbreeding and *K* is Queller and Goodnight (1989) relatedness. Reproduced from Fant *et al.*, 2013  Table 1, and unpublished data from J.B.F. (1997 and 2003). Summary means include standard errors. States are IL, Illinois; IN, Indiana; MI, Michigan, and WI, Wisconsin.

Site name^1^	Population size	Year	Average sampling effort (number of plants sampled)	*H*_e_	*F*_IS_	*K*
**Native population sites**						
Kohler Andrae, WI	4	2008	29.40 (50)	0.16	0.27	0.80
West Beach, IN	4	1997	10.86 (50)	0.25	0.18	0.43
West Beach, IN	4	2003	30.00 (50)	0.25	0.18	0.28
West Beach, IN	4	2008	44.57 (50)	0.23	0.10	0.52
West Beach, IN		**Mean**	28.48 ± 6.91	0.24 ± 0.02	0.15 ± 0.03	0.40 ± 0.11
Howes Prairie, IN	4	1997	11.29 (50)	0.19	0.00	0.42
Howes Prairie, IN	4	2003	31.29 (50)	0.24	0.36	0.34
Howes Prairie, IN	4	2008	38.14 (50)	0.37	0.33	0.21
Howes Prairie, IN		**Mean**	26.90 ± 8.05	0.27 ± 0.05	0.23 ± 0.11	0.28 ± 0.06
Big Blowout, IN	3	1997	11.86 (50)	0.32	0.06	0.29
Big Blowout, IN	4	2003	49.00 (50)	0.22	0.35	0.36
Big Blowout, IN	4	2008	36.57 (50)	0.34	0.23	0.30
Big Blowout, IN		**Mean**	32.48 ± 10.92	0.29 ± 0.04	0.21 ± 0.08	0.33 ± 0.02
Warren Dunes SP, MI	4	1997	17.00 (50)	0.25	0.46	0.34
Warren Dunes SP, MI	4	2003	40.86 (50)	0.33	0.16	0.32
Warren Dunes SP, MI	5	2014	− (13)	0.18	0.54	−
Warren Dunes SP, MI		**Mean**	28.93 ± 15.58	0.25 ± 0.04	0.39 ± 0.12	0.32 ± 0.01
Saugatuck Dunes SP, MI	4	1997	11.60 (50)	0.32	0.57	0.08
Saugatuck Dunes SP, MI	4	2003	30.00 (50)	0.27	0.39	0.21
Saugatuck Dunes SP, MI	4	2008	37.57 (50)	0.29	0.19	0.39
Saugatuck Dunes SP, MI	4	2014	− (20)	0.20	0.23	−
Saugatuck Dunes SP, MI		**Mean**	26.39 ± 12.82	0.27 ± 0.03	0.35 ± 0.09	0.39 ± 0.06
**All native sites**	4	**Mean**	23.35 ± 4.57	0.26 ± 0.01	0.27 ± 0.04	0.32 ± 0.04
**Reintroduction sites**						
Portage Lakefront Mid-successional	4	2009	15.86	0.52	0.37	0.02
Portage Lakefront Late-successional	4	2009	15.71	0.35	0.31	0.29
Portage Lakefront	4	**Mean**	34.30 ± 0.08	0.44 ± 0.09	0.39 ± 0.03	0.16 ± 0.14
Illinois Beach SP North	4	1997	27.70	0.44	0.31	0.13
Illinois Beach SP North	4	2008	16.60	0.33	0.16	0.29
Illinois Beach SP North	4	**Mean**	22.15 ± 5.55	0.39 ± 0.06	0.24 ± 0.08	0.21 ± 0.08
Illinois Beach SP South	4	1997	10.20	0.38	0.33	0.05
Illinois Beach SP South	4	2008	9.10	0.19	0.42	0.76
Illinois Beach SP South	4	**Mean**	9.65 ± 0.55	0.29 ± 0.10	0.38 ± 0.05	0.41 ± 0.36
**All reintroduction sites**	4	**Mean**	15.86 ± 2.70	0.37 ± 0.05	0.32 ± 0.04	0.26 ± 0.11

^1^The Howes Prairie site near the town of Dune Acres and the Portage Lakefront site near the town of Ogden Dunes were respectively called the latter town name in Fant *et al*. (2013): both are within the Indiana Dunes National Park, IN, USA.

Inbreeding coefficients in native and reintroduced populations remained high, averaging 0.27 ± 0.04 and 0.32 ± 0.04 respectively (*t*_15.6_ = −3.2, *P* = 0.006) (Table 3). In contrast, the relatedness (*K*) within populations was slightly lower in reintroduced sites, averaging 0.26 ± 0.11 compared with 0.32 ± 0.04 in native populations, but there were no significant differences between population types (*t*_6.5_ = 0.80, *P* = 0.44). The two lowest kinship values were in reintroduction populations: Illinois Beach SP South in 1997 (0.05) and Portage Lakefront mid-successional in 2009 (0.02; Table 3). The two highest kinship values were at native Kohler Andrae SP (0.80) and the reintroduction population at Illinois Beach SP South in 2008 (0.76). Interestingly, relatedness within the native populations remained similar over time but increased in the two Illinois Beach SP reintroductions over 11 years (1997–2008) (Table 3). Our study sites at Portage Lakefront mid- and late-successional populations (sampled in 2009) showed some of the highest genetic diversity (0.52 and 0.35 respectively) and lowest values for relatedness (0.02 and 0.29) compared with native populations, although with moderate inbreeding (0.37 and 0.31).

### Population expansion

**Fig. 7.**
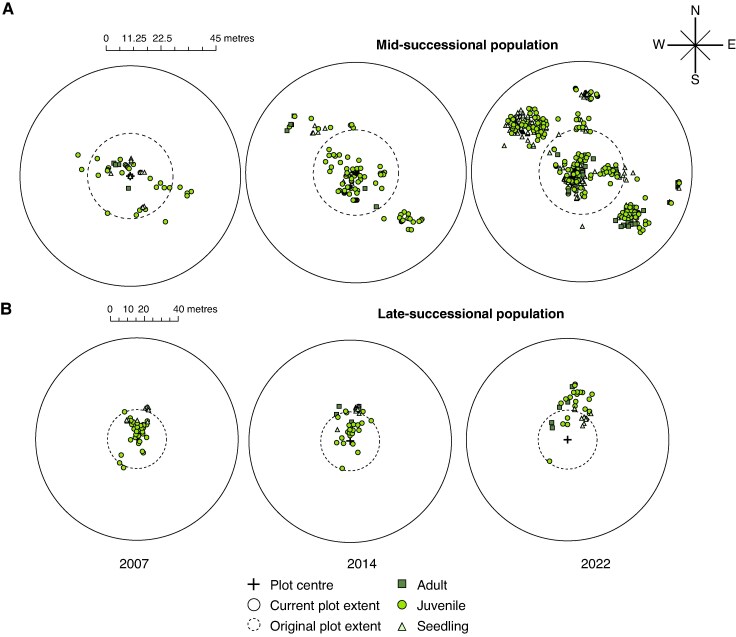
Pitcher’s thistle (*Cirsium pitcheri*) population expansion from initial planting plots at (A) the mid-successional (marram grass (*Ammophila breviligulata*)) site and (B) the late-successional (little bluestem (*Schizachyrium scoparium*)) site in 2007, 2014 and 2022, at Indiana Dunes National Park, IN, USA, including seedlings (triangles), juveniles (green circles) and reproductive adult plants (green squares). The inner dashed circle is the original tenth-hectare plot (17.84 m radius) and the solid circle is plot extent in 2022. Note the scale difference for A and B.

By 2007, plants had migrated outside the mid- and late-successional plots and have continued to do so (Fig. 7). The mid-successional population has been migrating both north-west and south-east, colonizing open patchy marram grass habitat while persisting in the central reintroduction area. Lakeward to the north-west, during the recent lake high stand of 2019–20, winter winds pushed sand inland, opening blowout habitat shoreward from the experimental plots. South-east of the mid-successional plot is the crest of the parabolic blowout where sand movement is prevalent due to wind exposure at higher elevations and due to loose sand from an unofficial trail. At the late-successional site, the population has been migrating from the original flat vegetated dune crest lakeward towards the north, where the large parabolic slope provides active sand movement from winter winds and slope slumping. Now all plants are found outside of the original experimental plots where little bluestem vegetation has remained stable.

## DISCUSSION

Population genetic techniques were not as advanced in 1994 as they are today and we did not have the current breadth of understanding of genetics issues in rare plant reintroduction (Frankham *et al.*, 2017). Nonetheless, we had the advantage of allozyme analyses (Loveless, 1984; Loveless and Hamrick, 1988), showing low genetic diversity across the Pitcher’s thistle range and experiments indicating much improved seed set with outcrossing. We also had the benefit of early metapopulation concepts of how gene flow might be structured across dynamic landscapes (Hanski, 1994; Pulliam, 1988) and a rich background illustrating phenomena of dune system succession developed in the Indiana Dunes by Henry Cowles (1899) and Jerry Olson (1958*a*, *b*).

These developing techniques and concepts, along with practical logistical considerations, inspired our approach to the thistle introductions, and our commitment to long-term demographic studies investigating whether out-planted populations can be self-sustaining. Genetic tools will continue to play an important role in the future management of Pitcher’s thistle or any rare plant, helping to understand evolutionary potential and resilience of native and restored populations (Frankham *et al.*, 2017; Thompson *et al.*, 2023; van Rossum *et al.*, 2023; Aitken *et al.*, 2024). This project demonstrates best practices for reintroducing Pitcher’s thistle, showing increased heterozygosity resulting from genetic mixing of local source populations, tracking the persistence of new populations from two one-time founder events with <2000 seeds each, demonstrating that these populations are resilient by dispersal into adjacent habitat and by developing techniques for planting reintroductions. This research could provide assistance to reintroductions of other disturbance-dependent monocarpic perennial plants (e.g. Pavlovic, 1994; Li *et al.*, 2020).

### Genetic mixing

Our mid- and late-successional reintroductions (Portage Lakefront) and those of Bowles and McBride (1996) at Illinois Beach SP produced populations with the highest mean number of alleles per locus, and expected heterozygosity compared with native populations in the southern Lake Michigan area (Fant *et al.*, 2013) (Table 3); thus, genetic mixing was successful as recommended and reported in other studies (St Clair and Howe, 2007; Willi *et al.*, 2007; ICUN, 2013; Frankham *et al.*, 2017; Center for Plant Conservation, 2019). Fant *et al.*’s (2014) and Sefton’s (2020) (Table 3) range-wide landscape genomic analyses of respectively seven and eight microsatellite loci demonstrated low genetic diversity and high inbreeding in the southern edge of the range. Fant *et al.* (2014) identified variation in the representation of four *K* clusters among six sites at the southern end of Lake Michigan, using the program STRUCTURE v2.2 (Pritchard *et al.*, 2000) (Fig. 4 in Fant *et al.*, 2014), illustrating how genetic mixing contributed to higher genetic variation in the reintroduced populations at Portage Lakefront. The declines in heterozygosity and increasing inbreeding coefficients at Warren Dunes and Saugatuck Dunes are consistent with the poorly documented decline of these populations in Michigan. While kinship was not different between native and reintroduced populations, the coefficients were lowest at the Portage Lakefront mid-successional population and Illinois Beach SP South reintroduction in 1997, respectively 15 and 6 years after establishment, this might reflect low flowering within patches and limited gene flow between patches within the restorations.

### Founder population size and rare plant reintroduction

The issue of founder population size has been an important topic for genetic rescue, rare plant reintroduction and conservation (Shaffer, 1981; Soule, 1986; Frankham *et al.*, 2017). Fant *et al.* (2014) found that population size was a stronger predictor of local Pitcher’s thistle genetic diversity than latitude or log of isolation. Maschinski and Albrecht (2022) suggest planting >50 whole plants, while van Rossum and Hardy (2022) suggest 700 transplants. Bell *et al.* (2003), with data from Illinois Beach SP, estimated from matrix modelling that 250 000 seeds or 1600 seedlings would be needed for establishment of a viable Pitcher’s thistle population. Bellis *et al.* (2023) found that large founder sizes reduced the probability of extinction and increased the probability of next-generation reproduction. Clearly, our reintroductions using ∼2000 seeds per site were fewer than some suggestions, and the two populations did experience steep declines in some years, but the populations have persisted. We hypothesize that in suitable habitats, such as Portage Lakefront, less than 250 000 seeds as Bell *et al.* (2003) suggested would be needed to establish a viable population.

Nevertheless, despite persistence, moderate genetic inbreeding suggests that more than a one-time founder event and introduction of juveniles would have benefited our reintroduction (Bell *et al.*, 2003; Bellis *et al.*, 2023). While the mid-successional population showed a large recent population expansion, the numbers of adults were < 22 (mean of 8 ± 1 adults per year) (Fig. 5). This census population size is much lower that the theoretical desired effective population size of > 50. Greater numbers of plants in these reintroductions would benefit the genetic viability of our populations (Frankham *et al.*, 2017). While high genetic diversity and low inbreeding are important for long-term population evolutionary potential (Forester *et al.*, 2022), population demographic viability is important as well (Knight, 2012). Halsey *et al.* (2016), using life table response experiments, showed positive population growth at the mid-successional site from 1999 to 2012 which did not include the population peak from 2017 to 2023 (Fig. 3). Thus, high inbreeding coefficients did not translate to poor demographic performance, at least over several decades in one of our reintroduced populations.

Frankham *et al.* (2017) and others have suggested that mixed mating plants, such as Pitcher’s thistle, would be likely to suffer from inbreeding depression, and have less potential for genetic rescue than fully outcrossing plants. Our results and those of others, including Dudash *et al*. (2024) in perennial *Mimulus* populations, counter the bimodal prediction of only selfing and outcrossing states and pose that stable mixed mating populations occur in nature. Perhaps Pitcher's thistle is less negatively impacted by inbreeding with low population sizes due to its history of population fluctuations, bottlenecks and long life history (Fant *et al.*, 2014). For example, Kohler Andrae SP in Wisconsin (which contributed seeds to the restoration at Illinois Beach SP (Bell *et al.*, 2002)), has low genetic diversity and high levels of inbreeding (Fant *et al.*, 2013, 2014; Table 3 and Fig. 6), but is persisting, despite having some of smallest plants and lower seed germination and survival (Staehlin and Fant, 2015). The persistence of our reintroductions with moderate inbreeding depression provides further evidence for stable mixed-mating populations and the role of epigenetic effects, environmental × genetic interactions and plasticity in contributing to population persistence as well (Carr and Dudash, 1995; Cheptou, 2024; Walker and Spigler, 2024).

Seedlings and plants have been the most used life stages for reintroductions rather than seeds, since planting seeds generally results in lower establishment rates (Albrecht and Maschinski, 2012). Sowing seeds eliminates the expense of growing plants in the greenhouse and out-planting them (Bowles *et al.*, 1993). However, development of a cohort-structured population can take longer when starting populations from seed (Bell *et al.*, 2003; Albrecht and Maschinski, 2012; Halsey *et al.*, 2017; Bellis *et al.*, 2023), and large quantities of seeds may be needed. In addition, the low establishment rates of broadcast versus planted seeds provide cautionary notes for a seeding approach. Mouse (*Peromyscus* spp.) predation of seeds lying on the ground was likely the cause for the broadcast failure, a result also seen by Loveless in field seeding trials (Loveless, 1984). The better survival of seedlings at the late-successional site compared with the mid-successional site early in the experiment was more likely due to a nurse plant effect (Maun 1994; Rand *et al.*, 2015; Landero and Valiente-Banuet, 2010 ) than to higher litter levels (Jolls *et al.*, 2015). The decline in seed germination with burial depth (Jolls *et al.*, 2015) may explain the higher germination in the broadcast compared with the sown plots at the early-successional site. Moderate sand burial over the 1994–95 winter thus did not prevent germination, but high burial in the winter of 1995–96 killed all the plants in the early-successional site irrespective of sowing method.

While a one-time seed sowing event was successful in establishing two populations that have persisted for 30 years, adding additional seeds or juveniles in subsequent years might have reduced the decline seen early in the experiment (Table 1), allowing the populations to attain a cohort-structured population sooner with a larger effective population size (Deredec and Courchamp, 2007), as also suggested by Frankham *et al.* (2017) and others. In addition, the mid-successional population, where all sown seeds were rated as perfect, fared better overall than the late-successional site, indicating that seed quality may play a role in seeding outcomes (e.g. Godefroid *et al.*, 2016). While Staehlin and Fant (2015) found geographic differences in germination success in Pitcher’s thistle seeds across the whole range of the species at the regional level, we did not find germination differences among the donor populations. Tardy and Godefroid (2024), in three rare perennial herbs in Belgium, found large differences in performance based on multiple sources of seeds used in reintroductions (54 donors per population among three source populations). These results point to the genetic benefits of admixture and repeated seed additions early in the reintroduction as a means of enhancing potential genetic diversity, producing larger effective populations to reduce the potential of inbreeding depression (St Clair and Howe, 2020). Successful reintroductions using a diversity of maternal lines to establish numerous large local populations may be important for range-wide Pitcher’s thistle conservation and for other rare plant species as well (Frankham *et al.*, 2017, 2019; Albrecht and Edwards, 2020; van Rossum *et al.*, 2023). Many studies suggest that genetic mixing is beneficial in reintroductions (e.g. Vergeer *et al.*, 2005; van Rossum and Le Pajolec, 2021); however, many other non-genetic factors may also impact population persistence, including translocation methods, disease and pests (Burli *et al.*, 2025) as well as variation in genetic diversity of a species across its natural range (e.g. Kaulfuß and Reisch, 2017).

### Indiana Dunes metapopulation dynamics

The fates of our reintroductions relative to site vegetation composition show that successional habitat variation is essential for maintaining Pitcher’s thistle populations. For example, based on population size, the late-successional site did more poorly in the more dense and stable dune vegetation than the mid-successional site, despite receiving an additional 600 seeds of lesser quality (McEachern, 1992; McEachern *et al.*, 1994). However, despite the late-successional population being less successful in terms of population size, it was able to migrate into more suitable adjacent early-successional habitat. Such migration of Pitcher’s thistle within and beyond the initial plots demonstrates its ability to move in the landscape tracking open sand and disturbed habitats over relatively short periods of time. Using 50-year projection models of metapopulation viability at the Indiana Dunes, Halsey *et al.* (2015, 2016) showed that both restoration introductions and local population enhancement can increase metapopulation viability, especially when the restorations included early- and mid-successional habitats.

### Building resilience to unexpected events

Thirty years of population persistence is more striking given that the populations experienced several unexpected events that reduced population growth and even threatened to destroy them. In 2010, eastern cottontail rabbits (*Sylvilagus floridanus*) consumed most of the leaves on Pitcher’s thistle juvenile plants at the late-successional site and in 2011 the mid-successional population was accidentally burned. Also in 2011, we discovered that the non-native root-boring weevil, *Cleonus pigra*, was killing large juveniles and bolting adults. The non-native seed-feeding weevils *Larinus carlinae* and *Rhinocyllus conicu*s are also present at the site (N. B. Pavlovic, pers. obs.). Hakes and Meunier (2018) and Gijsman and Vitt (2020) have documented weevil feeding decreasing Pitcher’s thistle seed production by respectively 50–80 and 60% at their study sites farther north at the Door Peninsula of Wisconsin. Such predation has the potential to drive the local Indiana Dunes population to decline and even extirpation (Havens *et al.*, 2012) even with our best efforts.

Land management decisions can have consequences for population longevity that could overwhelm natural phenomena (Bowles and McBride, 1996; Bowles and Bell, 1998; Bell *et al.*, 2002, 2013). In October 2008, an access road, parking lot and pavilion were built at the east side of the Portage Lakefront area, increasing public access with trampling and dune destabilization near our restoration plots. The construction of a house at the west end of the park in the town of Ogden Dunes in 2004 added to the loss of potential habitat. Finally, the jack pine stand nestled between the shoreline and our sites has been growing in stature and may eventually reduce wind velocity and sand movement, a process vital for Pitcher’s thistle persistence.

Each of these events was unexpected in 1994, but the lesson is to ‘expect the unexpected’. This means that we must create rare plant populations with exceptional resiliency via large population size, broad spatial representation and high genetic diversity at the outset for long-term persistence. As succession advances and visitation pressure increases in the dune habitats, novel management strategies may become more important for maintaining and conserving habitat mosaics. The National Park Service is looking into ways to protect the dunes from visitor access. Ironically, lessons learned from an accidental fire show that it might become a useful tool to arrest successional advancement and remove litter, a potential beneficial impact on Pitcher’s thistle in minimizing suitable habitat for the invasive weevils (Jolls *et al.*, 2015; Hakes and Meunier, 2018).

In conclusion, two of our single founder event reintroductions have proven to be viable for 30 years, and have demonstrated that sowing seed is preferable to broadcasting, and germination was not influenced by seed source. More importantly, genetic mixing of source populations was successful in producing populations with high genetic diversity and low kinship, genetic factors that probably contributed to population persistence, the ability to increase in population size and the ability of the population to migrate into adjacent suitable habitat. While inbreeding was moderate with low kinship values, especially at the mid-successional site, this lessened the concerns about the possible deleterious effects of inbreeding. Clearly, population viability would be improved with adding more plants generated from other seed sources to the population.

Perhaps the best contribution conservationists like us can contribute to long-term persistence is effective communication interpreting the results and knowledge gained through our science to help future generations of land managers conserve rare plant species populations and their habitats (Burli *et al.*, 2025). While syntheses of many reintroductions have provided great insights into best management practices (Bellis *et al.*, 2023, 2025), detailed long-term studies such as ours show best management practices for creating new populations and illustrate the combination of genetic, demographic and ecological information that can be important in evaluating reintroduction success. While synthesis studies document rare plant reintroductions of >30 years (e.g. Bellis *et al.*, 2023; longest duration was 46 years), few detailed studies have documented reintroductions of this duration and this work fills the gap of scant scientific documentation of such long duration reintroduction studies (Fenu *et al.*, 2023).

## Data Availability

The data release has three files. The first has the numbers of seeds planted by successional habitat and by stage from 1994 to 2000. The second and third files, of identical structure, have the demographic data for the mid- and late-successional site from 2001 to 2023. Each file presents the plant tag number, distance from the plot center, aximuth from the center to the plant and the stage (seedling, juvenile and adult) for each year that the plant was alive (see Pavlovic and McEachern, 2025b). https://doi.org/10.5066/P13RWVVU.
